# No Evidence of Abnormal Expression of Beta-Catenin and Bcl-2 Proteins in Pilomatricoma as One Clinical Feature of Tetrasomy 9p Syndrome

**DOI:** 10.1155/2021/2612846

**Published:** 2021-12-15

**Authors:** Chariyawan Charalsawadi, Sasipong Trongnit, Kanoot Jaruthamsophon, Juthamas Wirojanan, Somchit Jaruratanasirikul, Anupong Nitiruangjaras, Pornprot Limprasert

**Affiliations:** ^1^Department of Pathology, Faculty of Medicine, Prince of Songkla University, Songkhla, Thailand; ^2^Department of Pediatrics, Faculty of Medicine, Prince of Songkla University, Songkhla, Thailand; ^3^Faculty of Medicine, Siam University, Bangkok, Thailand

## Abstract

**Background:**

Little is currently known about the genetics of pilomatricoma. A number of studies have reported some evidence that this disease may have a genetic association with mutations of *CTNNB1* gene or expression of the beta-catenin protein. In this study, we reviewed literatures involving 30 patients with various genetic syndromes that have been linked to pilomatricoma and found that somatic mutations of the *CTNNB1* gene were reported in 67% of patients. Pilomatricoma has been reported in patients with chromosome 9 rearrangements, including 4 patients with tetrasomy 9p syndrome and one patient with partial trisomy 9. In addition to beta-catenin, the expression of bcl2 was observed in pilomatricoma.

**Objectives:**

To report an additional case of tetrasomy 9p syndrome with concurrent pilomatricoma and to examine whether abnormal protein expressions of the *CTNNB1* and/or *BCL2* genes were present.

**Methods:**

Cytogenetic analysis was carried out on peripheral blood, biopsied skin, and pilomatricoma tissue obtained from a patient with tetrasomy 9p syndrome. Immunohistochemical staining was performed on the pilomatricoma tissue, using beta-catenin and bcl2 monoclonal antibodies.

**Results:**

SNP microarray revealed nonmosaic gain of the short arm of chromosome 9. A nonmosaic isodicentric chromosome 9 was identified in the peripheral blood but this rearranged chromosome was detected in only 8.3% of the skin fibroblasts. Chromosomal abnormalities were not detected in the pilomatricoma nor expression of beta-catenin or bcl2 proteins in our patient.

**Conclusion:**

Pilomatricoma could be a new clinical feature associated with tetrasomy 9p syndrome; however, we found no evidence of tetrasomy 9p or abnormal beta-catenin or bcl2 proteins of the *CTNNB1* and *BCL2* genes in our pilomatricoma patient.

## 1. Introduction

Pilomatricoma, also known as pilomatrixoma or epithelioma calcificans of Malherbe (OMIM #132600), is the most common benign tumor of hair matrix cells. This usually occurs on the head or upper trunk in young people. Pilomatricoma usually occurs in solitary without other symptoms; multiple pilomatricomas have rarely been reported [1]. Pilomatricoma has been attributed to *CTNNB1* mutations in both nonsyndromic and syndromic cases. An association between pilomatricoma and some genetic conditions has been suggested, with tumorigenesis linked to genes responsible for these genetic conditions via beta-catenin regulation in the Wnt signaling pathways. A number of patients with pilomatricoma(s) in the presence of concurrent genetic conditions have been reported, including Gardner syndrome [2–4], Rubinstein-Taybi syndrome [5–10], myotonic dystrophy [11–16], Turner syndrome [17–22], Kabuki syndrome [23, 24], tuberous sclerosis [25, 26], constitutive mismatch repair deficiency [27], Sotos syndrome [28, 29], neurofibromatosis I [30], Stickler syndrome [22], MYH-associated polyposis [31], partial trisomy 9 [32], and tetrasomy 9p syndrome [33, 34]. The *CTNNB1* gene (OMIM∗116806) on chromosome 3p22.1 encodes beta-catenin, a 92 kD cytoplasmic protein that plays an important role in the creation and maintenance of epithelial cell layers by regulating cell growth and adhesion between cells. Activated mutations in *CTNNB1* leading to upregulation of beta-catenin have been observed in pilomatricomas [35, 36] and pilomatrical carcinomas [35], especially mutations in exon 3 of the gene which have been observed in approximately 60-75% of pilomatricomas [36]. Beta-catenin is stabilized by Wnt signaling and is associated with the TCF/LEF family of transcription factors, thus activating transcription of Wnt target genes [37]. The Wnt signaling pathways play a crucial role in both normal developmental processes as well as in tumorigenesis of many types of neoplasia. Studies have shown that beta-catenin is involved with hair follicle development and stem cell differentiation in the skin [38]. In addition to beta-catenin protein, the expression of bcl2 protein, which is an antiapoptotic protein encoded by the *BCL2* gene (OMIM +151430) on chromosome 18q21.33, was observed. It was suggested that the faulty suppression of apoptosis contributes to the pathogenesis of pilomatricoma [39].

In this article, we present an additional case of pilomatricoma concurrent with tetrasomy 9p syndrome. Tetrasomy 9p syndrome is a rare chromosomal disorder caused by the presence of an extra isochromosome of the short arm of chromosome 9. The phenotypes of this syndrome vary widely, from stillborn babies with multiple congenital malformations to normal healthy adults. Some notable clinical features of the syndrome include prenatal and postnatal growth retardation, delayed developmental milestones, and craniofacial dysmorphism. Craniofacial dysmorphisms associated with this syndrome are asymmetric skull shape, downslanting palpebral fissures, hypertelorism, epicanthal folds, microphthalmia or enophthalmia, prominent nasal bridge, bulbous or beaked nose, large mouth with down-turned corners, cleft lip, cleft palate, malformation and/or malposition of the ears, brachycephaly or microcephaly, wide cranial sutures, large fontanelle, micrognathia, short neck, excess nuchal skin, congenital heart defects, urogenital defects, abnormalities of central nervous system (e.g., Dandy-Walker malformation and hydrocephalus), and hand or foot deformities (e.g., flat feet, club feet, rocker bottom feet, hypoplastic or dysplastic fingers, toes, and nails) [34]. Pilomatricoma has been reported in patients with gain of chromosome 9, including 4 cases with tetrasomy 9p syndrome and one case with partial trisomy 9 [32–34]. The association between gain of the short arm of chromosome 9 and development of pilomatricoma is still unclear and has never been studied. In this study, we performed genetic analysis of the pilomatricoma in our patient and also reviewed the literature regarding gene and chromosomal mutations associated with pilomatricoma.

## 2. Clinical Report

**Figure 1:**
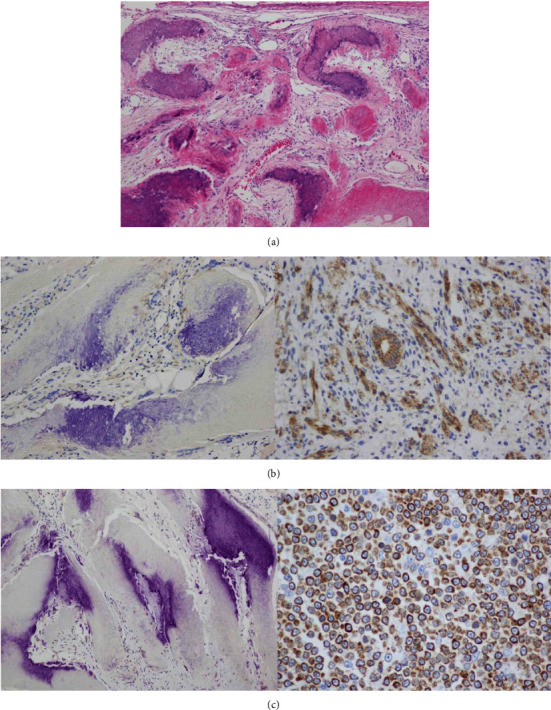
Histological findings of pilomatricoma in our patient. (a) H&E staining revealed a tumor composed of basaloid cells, ghost or anucleated shadow cells, and areas of cystic degeneration. The basaloid cells are darkly stained, round, or elongated with deeply basophilic nuclei. The ghost or anucleated shadow cells have abundant pale, eosinophilic cytoplasm with a clear central area. The latter are highly characteristic of pilomatricoma. (b) Immunohistochemical staining of beta-catenin in the patient's pilomatricoma tissue showed no beta-catenin expression (left) and positive control (right). (c) Immunohistochemical staining of bcl2 in the patient's pilomatricoma tissue showed no bcl2 expression (left) and positive control (right).

Our patient was a 4-year-old girl, the only child of nonconsanguineous parents. The mother was 38 years old, and the father was 36 years old at the time of the patient's birth. There was no family history of congenital malformation. Amniocentesis for prenatal diagnosis was not done. She was born at term by spontaneous vaginal delivery, with birth weight of 2,735 g (between the 3^rd^ and 10^th^ centiles) and length of 51 cm (50^th^ centile). She was first brought to the pediatric clinic at 11 months of age due to developmental delay. Gross motor development and generalized hypotonia were noted. An MRI brain at the age of one year revealed prominent choroid plexuses in ventricular system and diffused dilatation of the ventricular system with indications of some white matter loss. The cerebral cortex, corpus callosum, cerebellum, pituitary, and myelination were normal. Sensory and motor nerve conduction tests of the lower extremities showed no abnormalities. Physical examination at 4 years old revealed dysmorphic features of frontal bossing, epicanthal folds, high-arched palate, oligodontia, transverse palmar crease on the left hand, clinodactyly on the right hand, foot deformities (pes planus, heel valgus, and subluxation of the proximal interphalangeal joints), hypermobile joints, and generalized abnormal skin pigmentation over the trunk and extremities. An abdominal mass near the umbilical area was first noticed when the patient was 2 years old, and it had gradually grown to approximately 3 cm in diameter by the age of 4 years. The mass was round, firm, and well defined, with overlying abnormal skin pigmentation. It was not itchy or painful. The mass was excised and sent for histopathological examination. Histopathological examination of the tumor showed findings on hematoxylin and eosin (H&E) staining that were consistent with pilomatricoma (Figure 1(a)). At 5 years old, physical examination revealed a small round, firmed, and well-defined mass (approximately 0.3 cm in diameter) at the back of neck but it was not excised. She was also tested with Stanford Binet intellectual scale (5^th^ edition), which revealed mild intellectual disability (full scale IQ = 64, nonverbal IQ = 66, and verbal IQ = 66).

## 3. Materials and Methods

Cell culture was performed on peripheral blood. Subsequent G-banding karyotype analysis revealed an extra structurally abnormal chromosome (ESAC). Single nucleotide polymorphism (SNP) microarray was utilized to identify the origin of the ESAC. Briefly, we extracted DNA from peripheral blood using a FlexiGene ® DNA kit (Qiagen, Hilden, Germany) following the manufacturer's protocol. An Illumina HumanCytoSNP-12 v2.1 array was subsequently carried out according to the manufacturer's protocol (Illumina Inc., San Diego, CA). A total of ∼300,000 SNPs were analyzed using the Illumina GenomeStudio software v2011.1. The microarray findings were confirmed by fluorescence *in situ* hybridization (FISH) on metaphase spreads using subtelomeric probes specific to the short arm of chromosome 9 (RP11-174 M15) and the long arm of chromosome 9 (RP11-885 N19). To examine whether pilomatricoma tissue from our patient contained any chromosomal abnormalities, G-banding karyotype analysis was carried out on pilomatricoma tissue and cultured skin fibroblasts overlying the tumor.

To examine whether there was abnormal beta-catenin expression in the pilomatricoma of our patient, we carried out immunohistochemical staining using the beta-catenin (14) mouse monoclonal antibody (Cell Marque, Sigma-Aldrich, USA) on paraffin-embedded tumor tissues following the manufacturer's protocol. In addition, we used the bcl2 (100/D5) mouse monoclonal antibody (Leica Biosystem, USA) to study the expression of the bcl2 protein. We used a BOND-MAX automated IHC/ISH Stainer (Leica Biosystems, IL, USA) for immunohistochemical staining. We performed positive and negative tissue controls to examine whether all reagents were function properly, and color-reaction product was seen and unseen within target cells, respectively. Beta-catenin protein is normally found in the cytoplasm of the submembranous location. Mutations in the gene result in nuclear accumulation of the protein. For bcl2 protein, staining pattern is seen in membrane and/or cytoplasmic.

## 4. Results

**Figure 2:**
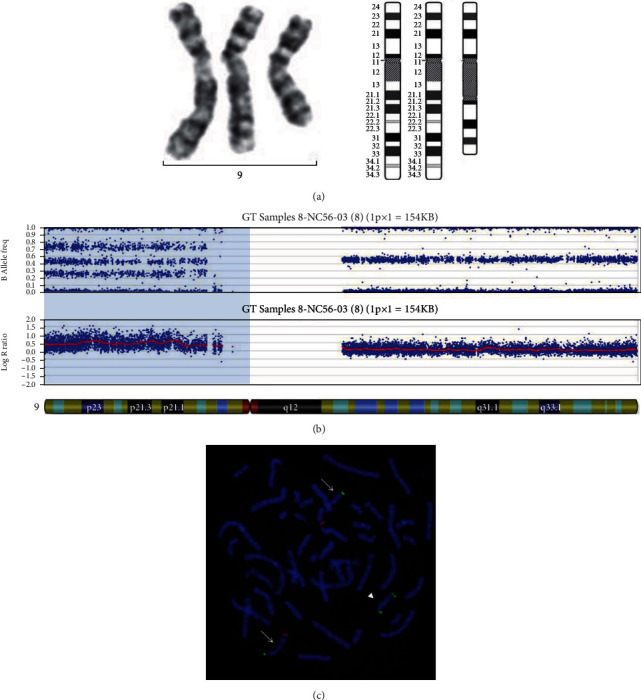
G-banding karyotype, FISH, and SNP microarray findings in the study patient. (a) Partial G-banding karyotype of chromosome 9 showing two normal chromosomes 9 and an isodicentric chromosome 9p (left) and ideogram of chromosome 9 (right). (b) SNP microarray analysis results showing an allele frequency graph (upper panel) and a log *R* ratio graph (lower panel). Note that altered B allele frequency consistent with 4 copies of the short arm of chromosome 9, relative to 2 copies of the long arm of chromosome 9, and elevated log *R* ratio graph for the short arm of chromosome 9. (c) FISH showing two normal chromosomes 9 (arrow) with a green signal of subtelomeric probe specific to the short arm and a red signal on the long arm of each chromosome 9. The isodicentric chromosome 9p (arrowhead) with two green signals of subtelomeric probe specific to the short arm of chromosome 9 on both ends of the chromosome.

Tetrasomy 9p syndrome was diagnosed in our patient by a combination of G-banding karyotype analysis on peripheral blood and SNP microarray. An ESAC that likely contained the short arm of chromosome 9 (Figure 2(a)) was identified by G-banding karyotype analysis in all cultured lymphocytes (20/20 cells). Then, DNA extracted from uncultured peripheral blood was analyzed on peripheral blood to further characterize the ESAC. The results obtained from the SNP microarray revealed nonmosaic gain of the short arm of chromosome 9 (Figure 2(b)). The gain of the short arm of chromosome 9 was confirmed by FISH (Figure 2(c)). The ESAC was a pseudoisodicentric of the short arm of chromosome 9. Parental karyotypes showed normal chromosomal complements. Her karyotype designation was 47,XX,+psu idic(9)(q12).arr [GRCh37] 9p24.3p12(46,587_42,374,011)x4 dn. By G-banding karyotype analysis, mosaicism for tetrasomy 9p was observed in the cultured skin fibroblasts (3/36 cells), but not in the pilomatricoma tissue (0/27 cells). No beta-catenin (Figure 1(b)) nor bcl2 (Figure 1(c)) proteinn expression was observed on the basaloid cell or transitional cell components of the pilomatricoma by immunohistochemical analysis. Positive reactivity was also shown in Figures 1(b) and 1(c).

## 5. Discussion

It is well established that genetic factors play a significant role in tumorigenesis in many types of neoplasias. The genetic processes underlying pilomatricoma are currently not well understood. Various studies have reported that *CTNNB1* gene mutations in pilomatricoma and pilomatrix carcinoma. The *CTNNB1* gene encodes the protein beta-catenin that is implicated in the Wnt pathway. Moreover, mutations in exon 3 of the gene that result in increased expression of intracytoplasmic and intranuclear beta-catenin result in hair matrix neoplasia development [40, 41]. There are 30% to 100% of pilomatricomas are associated with *CTNNB1* gene mutations [40, 42, 43]. The other researchers discovered the bcl-2 oncogene overexpression in pilomatricoma basophilic cells [39].

In nonsyndromic cases, pilomatricoma occurs mostly as a solitary tumor, whereas in syndromic cases, pilomatricoma often occurs as multiple tumors. We reviewed the reports of 30 patients with various genetic syndromes who developed pilomatricoma(s). Twelve patients, including 2 pairs of siblings, had available information regarding mutations of the *CTNNB1* gene or expression of the beta-catenin protein. Multiple pilomatricomas were observed in the majority of patients (80%). Solitary tumors were observed in patients with various chromosomal syndromes, including 5 patients with Turner syndrome. Four patients with tetrasomy 9p syndrome and a patient with partial trisomy 9 had multiple tumors. Mutations of the *CTNNB1* gene or expression of the beta-catenin protein were found in 8 patients (67%), whereas 4 patients had no mutations nor beta-catenin protein expression (33%). All patients with mutations of the *CTNNB1* gene or expression of beta-catenin protein had multiple tumors. Recurrent point mutations included c.122C>T (p.Thr41Ile), c.121A>G (p.Thr41Ala), and c.97 T>C (p.Ser33Pro) in exon 3 of the *CTNNB1* gene. A point mutation, c.122C>T (p.Thr41Ile), was also reported in patients with multiple pilomatricomas without any associated genetic conditions [44]. Mutations found in patients with genetic conditions and concurrent pilomatricomas are reviewed in Supplementary Table 1. By using immunohistochemical staining, nuclear beta-catenin staining was observed in 81% of pilomatricomas [36]. In one study, the beta-catenin expression was observed in both the nuclei and membranes of basaloid cells, but not in the transitional or shadow cells of pilomatricoma tissue [45]. However, another study found beta-catenin expressed strongly in transitional cells, but not in basophilic or shadow cells [46]. In another study, in addition to *CTNNB1* mutations and beta-catenin protein expression analyses, cytogenetic analysis of pilomatricoma tissues was carried out and revealed that approximately 60% of pilomatricomas had trisomy 18 in very low proportions varying from 0.5% to 2.8%, in the neoplastic epithelial cells of the pilomatricomas, but not in the surrounding mesenchymal or epithelial tissue [47].

In this study, we found that beta-catenin and bcl-2 protein were not expressed in the pilomatricoma tissue obtained from our patient. We did not find an isodicentric short arm of chromosome 9 nor other chromosomal abnormalities in the pilomatricoma tissue. Selection against cells with structurally abnormal chromosome 9 during the cell culture may have attributed to the negative finding in our patient. Although isodicentric of the short arm of chromosome 9 was not observed, we could not rule out the possibility of low-level mosaicism for the structurally abnormal chromosome 9, as mosaicism is a common phenomenon in tetrasomy 9p syndrome. SNP microarray using DNA from uncultured pilomatricoma tissue is a useful method that may overcome issues from cell culture selection. SNP microarray also has advantages as it allows for analysis of millions of cells, which is useful for the detection of low-level mosaicism. In addition, loss of heterozygosity, which is biologically equivalent to the second hit in the Knudson hypothesis, can also be detected by SNP microarray. Therefore, SNP microarray could be a valuable tool to further investigate whether gain of chromosome 9 material is associated with pilomatricoma. In our patient, we could not perform SNP microarray on DNA extracted from the paraffin-embedded pilomatricoma tissue due to very poor DNA quantity and quality.

Pilomatricoma is relatively common in the pediatric population, and therefore, we are hesitant to conclude that the pilomatricoma in our case was associated with tetrasomy 9p syndrome and could be simply an incidental finding in our case. However, as there are a few reported cases of tetrasomy 9p syndrome and partial trisomy 9 with multiple pilomatricomas and other genetic syndromes that predispose to the development of pilomatricomas, it is of interest to examine potential associations between these genetic syndromes and the development of pilomatricomas. Several genes located on the short arm of chromosome 9 that have been shown to be associated with tumorigenesis, such as BCL2-associated athanogene 1 (*BAG1*) at 9p13.3, cyclin-dependent kinase inhibitor 2A (*CDKN2A*) at 9p21.3, cyclin-dependent kinase inhibitor 2B (*CDKN2B*) at 9p21.3, nuclear factor I B (*NFIB*) at 9p23-p22.3, programmed cell death 1 ligand 1 (*PD-L1*) at 9p24.1, Janus kinase 2 (*JAK2*) at 9p24.1, programmed cell death 1 ligand 2 (*PDCD1LG2*) at 9p24.1, and SWI/SNF Related, Matrix Associated, Actin Dependent Regulator Of Chromatin, Subfamily A, Member 2 (*SMARCA2*) at 9p24.3. Mutations of *CDKN2A*gene have been reported in different types of benign and malignant tumors, including skin tumors [48]. According to the database, *CDKN2A* and *CTNNB1* genes are known to have interactions from both curated database and experimental database (Supplementary Figure 1). There was evidence that the expression of CDKN2A protein was frequently silenced in tumors through promoter hypermethylation. This suggested that CpG methylation changes may be induced by oncogenic beta-catenin, where beta-catenin was found to have interacted with DNA methyltransferase I in a mutually stabilizing interaction in the nuclei of cancer cells [49]. Although, majority of *CDKN2A* mutations are inactivating mutations (i.e., loss-of-function mutations), an interesting study found an increased copy number of *CDKN2A* gene in a nonsyndromic case with cutaneous pilomatrical carcinosarcoma but mutation of exon 3 of the *CTNNB1* gene was not detected in that case [50]. For the other genes on the short arm of chromosome 9, there were no evidences that link them to pathogenesis of pilomatricoma.

## 6. Conclusion

We reported pilomatricomas in an additional patient with tetrasomy 9p syndrome. Pilomatricoma could be a new clinical feature associated with tetrasomy 9p syndrome; however, we found no evidence of tetrasomy 9p or abnormal beta-catenin or bcl2 proteins of the *CTNNB1* and *BCL2* genes in pilomatricoma of our patient. We suggest further studies using collections of patients with genetic syndromes with concurrent pilomatricomas to examine potential associations between genetic syndromes and the development of pilomatricomas and to better understanding and insights into the mechanisms driving the growth and homeostasis of the human hair follicle.

## Data Availability

Data is available at a reasonable request from the corresponding author.

## Supplementary Materials

Supplementary Materials Supplementary Table 1 was included to provide the reader with further information regarding mutations found in patients with genetic conditions and concurrent pilomatricomas. Supplementary Figure 1: Gene network shows interactions between *CTNNB1* gene and other genes, including *CDKN2A* gene on chromosome 9 (https://string-db.org/).
