# Supplementary material for: No Evidence of Abnormal Expression of Beta-Catenin and Bcl-2 Proteins in Pilomatricoma as One Clinical Feature of Tetrasomy 9p Syndrome
Source: Int J Pediatr. 2021 Dec 15;2021:2612846. doi: 10.1155/2021/2612846 (PMC8694977; doi:10.1155/2021/2612846)

Supplementary Table 1. Mutations found in patients with genetic conditions and concurrent pilomatricomas[†]

Reference	Patient			Germline mutation	Somatic mutation
	**Age (years)**	**Gender**	**Number of pilomatricomas**		
**Gardner syndrome**					
Trufant et al., 2012 [3][††]	12	Male	Multiple	2 bp deletion (c.426_427delAT) in exon 4 of *APC* gene → frameshift (p.Ser142fs*146)	Lower nuclear beta-catenin protein in basaloid component than in sporadic pilomatricomas
	14	Female	Multiple		
Bendelsmith et al., 2018 [4]	11	Female	Multiple	Duplication (c.595dupG) in *APC* gene → frameshift (p.Ala199Glyfs*53)	NA
**MYH-associated polyposis**					
Baglioni et al., 2005 [31][††]	32	Male	Multiple	Homozygous 2 bp insertion (c.1186_1187insGG) in exon 13 of *MYH* gene → premature stop codon at position 438	No mutations in exon 3 of *CTNNB1* gene
	29	Female	Multiple		
**Rubinstein-Taybi syndrome**					
Rokunohe et al., 2016 [9]	12	female	Multiple	Duplication (c.5837dupC) in exon 31 of *CREBBP* gene → frameshift (p.Pro1947Thrfs*19)	c.122C>T (p.Thr41Ile) at phosphorylation site of beta-catenin protein
Papathemeli et al., 2015 [8]	49	Female	Multiple	Point mutation (c.6127C>T) in exon 31 of *CREBBP* gene → nonsense (p.Glu2043*)	NA
**Turner syndrome**					
Maeda et al., 2014 [21]	22	Female	Multiple	NA	Expression of beta-catenin protein in nuclei of basaloid cells
Handler et al., 2013 [20]	5	Female	Solitary	45,X	NA
	15	Female	Solitary	45,X/46,X,idic(X)	NA
	24	Female	Multiple	45,X	NA
	16	Female	Multiple	45,X	NA
	11	Female	Multiple	46,X,del(X)(p11.22)	NA
	9	Female	Multiple	45,X/46,X,idic(Y)(p11.3)/47,X,idic(Y)(p11.3),idic(Y)(p11.3)	NA
	10	Female	Solitary	45,X/46,X,r(X)	NA
	4	Female	Solitary	45,X/46,X,psu dic(Y)(p11.3)	NA
Bengtzen et al., 2009 [19]	13	Female	Multiple	45,X/46,XY	NA
Noguchi et al., 1999 [17]	23	Female	Solitary	45,X	NA
	19	Female	Multiple	45,X/46,X,i(Xq)	NA

Supplementary Table 1. Mutations found in patients with genetic conditions and concurrent pilomatricomas†

Reference	Patient			Germline mutation	Somatic mutation
	Age (years)	Gender	Number of pilomatricomas		
**Sotos syndrome**					
Gilaberte et al., 2008 [28]	9	Male	Multiple	Deletion (A6442delAGCGACCA, Lys2151fs*) in exon 22 of *NSD1* gene	Same deletion of *NSD1* gene<br>No mutations of *CTNNB1* gene
**Constitutional mismatch repair deficiency**					
Chmara et al., 2013 [27]	11	Male	Multiple	Homozygous mutation, c.1164delT in exon 11 of *PMS2* gene → premature stop codon (p.His388Glnfs*9)	- No microsatellite instability in pilomatricoma tissues<br>- Loss of nuclear PMS2 protein expression in neoplastic and non-neoplastic cells in pilomatricoma tissues<br>- c.94G>T (p.Asp32Tyr), c.122C>T (p.Thr41Ile), c.121A>G (p.Thr41Ala), c.97T>C (p.Ser33Pro) in exon 3 of *CTNNB1* gene
	4	Female	Multiple	Maternal deletion of exons 7–8 c.(?_706)_(9031_?)del<br>and paternal a deletion of exons 9–15 c.(?_904)_(*+_?)del of *PMS2* gene	- No microsatellite instability in pilomatricoma tissues<br>- Loss of nuclear PMS2 protein expression in neoplastic and non-neoplastic cells in pilomatricoma tissues<br>- c.122C>T (p.Thr41Ile) in exon 3 of *CTNNB1* gene
	15	Male	Multiple	Heterozygous splice site mutation, c.705+2T>C in *PMS2* intron 6 and heterozygosity for 3 exonic polymorphic variants, including c.780C>G, c.1408C>T, and c.1621A>G	- c.122C>T (p.Thr41Ile), c.121A>G (p.Thr41Ala), c.97T>C (p.Ser33Pro) in exon 3 of *CTNNB1*gene
**Myotonic dystrophy**					
Rübben et al., 2020 [11]	39	Male	Multiple pilomatricomas and one pilomatrical carcinoma	> 400 CTG-repeat expansion in *DMPK* gene<br>Heterozygous mutation c.8494C>T (p.Arg2832Cys) in exon 3 of *ATM* gene	p.Ser33Cys, p.Ser33Phe, p.Gly34Val, p.Thr41Ile and p.Gly34dup of *CTNNB1* gene<br>No abnormal microsatellites and no mutations of mismatch repair genes (*MLH1, MSH2, MSH6,* and *PMS2*)<br>Heterozygous mutation c.8494C>T (p.Arg2832Cys) in *ATM* gene
**Gain of chromosome 9 materials**					
Matsuura et al., 2002 [33]	4	Female	Multiple	47,XX, +mar[22]/46,XX[8].ish i(9)(p10)(wcp9+) i(9) in 26.7% of cultured lymphocyte	NA

Supplementary Table 1. Mutations found in patients with genetic conditions and concurrent pilomatricomas†

Reference	Patient			Germline mutation	Somatic mutation
	Age (years)	Gender	Number of pilomatricomas		
Blaya et al., 2009 [32]	15	Male	Multiple	46 XY (42.4%)⁄47 XY + del9 (pter-q32) (57.6%)††† +del(9) in 57.6% of cultured lymphocyte	NA
El Khattabi et al., 2015 [34]	18	Female	Multiple	47,XX,+psu idic(9)(pter→q21::q21→pter)<br>idic(9) in 90% of cultured lymphocytes, 30% in cultured skin fibroblasts and 30% in urine sediment	NA
	7.5	Female	Multiple	47,XY,+psu idic(9)(pter→q12 or 13::q13 or 12→pter)<br>idic(9) in 90% of cultured lymphocytes	NA
	18	Female	Multiple	47,XY,+psu idic(9)(pter→q12 or 13::q13 or 12→pter)<br>idic(9) in 75% of cultured lymphocytes, 0% in cultured skin fibroblasts and 40% in buccal smear	NA
Our patient	4	Female	? Multiple	47,XX,+psu idic(9)(q12).arr [GRCh37] 9p24.3p12(46,587_42,374,011)x4 dn<br>100% of idic(9) in of cultured lymphocytes (20/20 cells), 8.3% of idic(9) in cultured skin fibroblasts (3/36 cells)	0% of idic(9) in pilomatricoma (0/27 cells)<br>No beta-catenin and bcl2 protein expression in basaloid component

† Only patients with information regarding mutations are included in the table. Association between pilomatricoma and some genetic conditions have been suggested; wherein, tumorigenesis has been linked between genes responsible for these genetic conditions in addition to beta-catenin regulation in Wnt signaling pathways. In Wnt signaling pathways, adenomatous polyposis coli, axin1, casein kinase 1 alpha, and glycogen synthase kinase 3 beta proteins are components of beta-catenin destruction complex. The complex binds to beta-catenin, and sequentially phosphorylate beta-catenin, targeting it for ubiquitin-dependent degradation by the proteasome [37]. Mutations of *APC* genes, which are responsible for Gardner syndrome, result in truncated APC proteins, and ultimately lead to an accumulation of beta-catenin protein [3]. Linking between genes responsible for other genetic conditions and pilomatricoma have been hypothesized with some supporting evidences. For example, mutations of *CREBBP* gene on chromosome 16p13.3 are responsible for Rubinstein Taybi syndrome. *CREBBP* gene plays a role in the maintenance of genomic integrity in stem cells, and initiation of a differentiation by controlling the interaction of the nuclear receptor family with the Wnt signaling cascade [a]. In tuberous sclerosis complex, mutations of *TSC1* and *TSC2* genes on chromosome 9q34 and 16p13.3, respectively, are responsible for the disease. TSC1/TSC2 protein complex is associated with glycogen synthase kinase 3 beta and axin1 proteins, which in turn, promotes beta-catenin degradation. Missense mutation of *TSC* gene caused its failure to inhibit beta-catenin [26, b]. In Turner syndrome, low levels of glypican-3 protein, encoded by *GPC3* gene on chromosome Xq26.2, results in increases of beta-catenin [20]. In constitutive mismatch repair deficiency, *CTNNB1* gene is a target for mutations when mismatched repair is impaired due to biallelic *PMS2* mutations [27].

References

a. M. Ono, K.K.Y. Lai, K. Wu, et al., "Nuclear receptor/Wnt beta-catenin interactions are regulated via differential CBP/p300 coactivator usage." *PLOS ONE*. vol. 13, no. 7, pp. e0200714, 2018.

b. B.C. Mak, H.L. Kenerson, L.D. Aicher, et al., "Aberrant beta-catenin signaling in tuberous sclerosis." *The American Journal of Pathology*. vol. 167, no. 1, pp. 107–116, 2005.

†† Study of siblings with the same genetic conditions.

†† Cytogenetic nomenclature remains the same as in the publication.

Supplementary Figure 1. Gene network shows interactions between *CTNNB1* gene and other genes, including *CDKN2A* gene on chromosome 9 (https://string-db.org/).

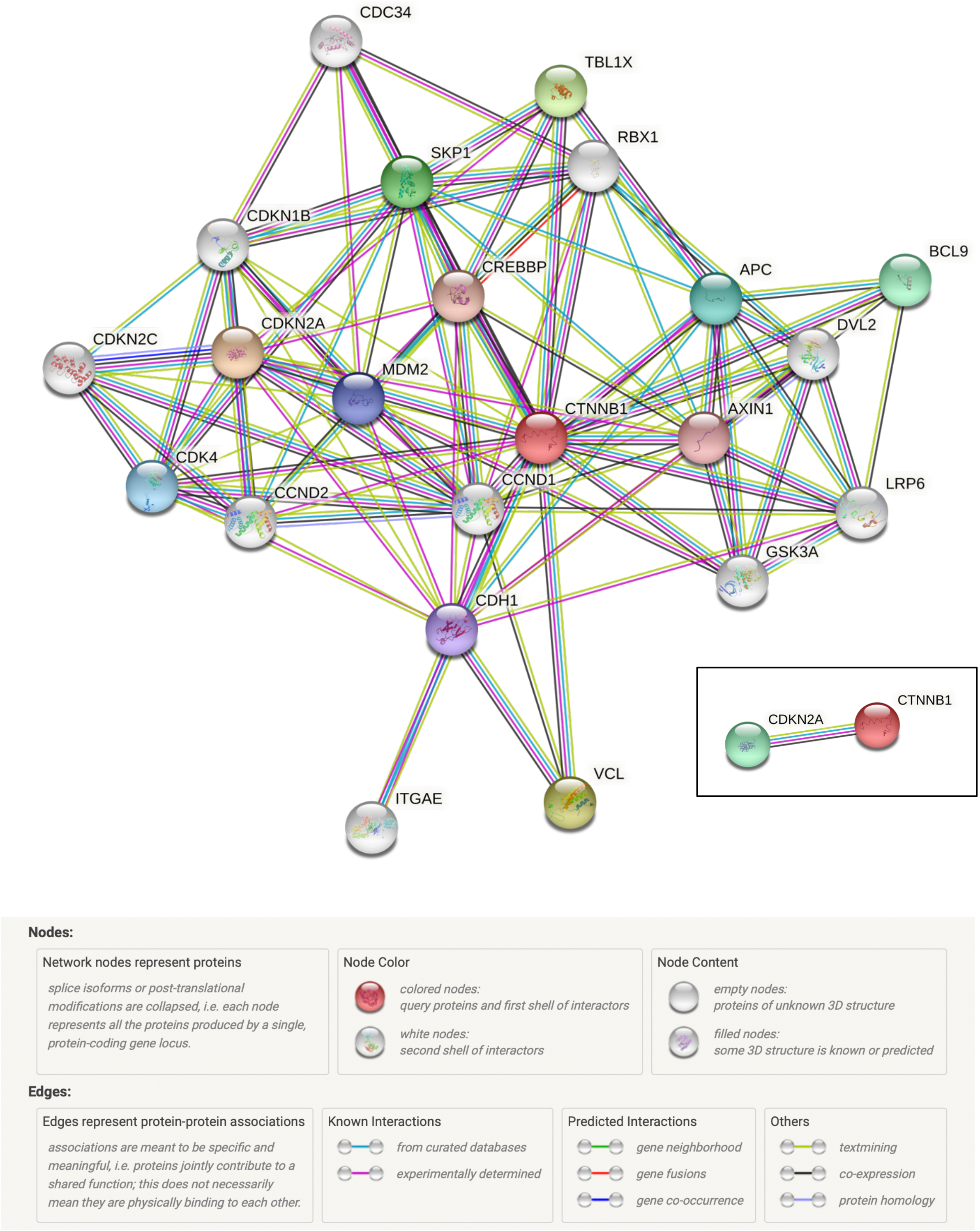

Supplement: Supplementary Materials — Supplementary Table 1 was included to provide the reader with further information regarding mutations found in patients with genetic conditions and concurrent pilomatricomas. Supplementary Figure 1: Gene network shows interactions between CTNNB1 gene and other genes, including CDKN2A gene on chromosome 9 (https://string-db.org/). [file 2612846.f1.pdf]
